# Community Health Participatory interventions in the prevention and control of non-communicable diseases including mental health in crisis-affected Low-and Middle-Income Countries – a scoping review

**DOI:** 10.1080/16549716.2025.2599011

**Published:** 2026-01-02

**Authors:** Sara Imtiaz, Nadia Khaleeq, Noor Sanauddin, Saima Afaq, Hannah Maria Jennings, Amber Tahir, Mariam Abdeali, Zala Khan, Farman Khan, Rubia Zafar, Asima Khan, Abdul Rahman Shahab, Farhad Ali, Helen Elsey, Farwah Hassan, Alishba Khan, Sayed Murtaza Sadat Hofiani, Nagina Alimi, Shabnam Azizi, Najeeb Alizoi, Khalid Rahman, Aziz Sheikh, Abdul Basit, Zia Ul Haq

**Affiliations:** a Public Health, Health Promotion Foundation, Karachi, Pakistan; b School of Public Health, Khyber Medical University, Peshawar, Pakistan; c Department of Sociology, University of Peshawar, Peshawar, Pakistan; d Department of Health Sciences, University of York, York, UK; e Global Mental Health, Hull York Medical School, York, UK; f Public Health, HealthNet TPO, Kabul, Afghanistan; g Nuffield Department of Primary Care, University of Oxford, Oxford, UK; h Usher Institute, University of Edinburgh, Edinburgh, UK

**Keywords:** conflict, hazards, empowerment, barrier, facilitators

## Abstract

The rising burden of Non-Communicable Diseases (NCDs) requires a comprehensive strategy by integrating community-based interventions – especially in Low- and Middle-Income Countries (LMICs). Over the past decade, researchers have emphasized communities as key agents of change in health systems. While Community Health Participatory (CHP) interventions show promise in NCD management, their application in crisis-affected contexts remains underexplored. This scoping review examines the adoption of CHP interventions, strategies employed, their barriers and facilitators encountered in crisis-affected LMICs to prevent and control NCDs. Utilising the Arksey and O’Malley framework, comprehensive search was conducted across PubMed, Web of Science, Scopus, and Google Scholar. Primary studies and grey literature in English were included focusing on CHP interventions among adults in such settings. Studies on unrelated health issues, review articles, protocols, and conference abstracts were excluded. Data extraction was conducted using Covidence, with discrepancies resolved through consensus. The narrative analysis of the extracted data was conducted. The review identified varied CHP interventions, with the majority focusing on mental health. The included studies highlighted the role of community engagement and stakeholders’ involvement. Strategies included raising awareness, providing social support and focusing on lifestyle modifications. Barriers to interventions included limited resources, socio-cultural constraints, and logistical challenges, while facilitators involved community leadership and ownership, empathy, cultural adaptations of interventions, and multi-sectoral collaboration.

CHP interventions represent a promising strategy for tackling NCDs in crisis-affected LMICs, however, limited evidence on their long-term impact needs further research.

This review was registered on the Open Science Framework and funding was provided by NIHR-UK (NIHR203248).

## Paper Context

- **Main finding:** Community Health Participatory interventions are increasingly applied in both urban and rural settings, utilizing varied strategies while maintaining community involvement as a core element to enhance empowerment for Non-Communicable Disease prevention and control.
- **Added knowledge:** This review underscores the emerging focus on Non-Communicable Disease prevention and control in crisis-affected Low- and Middle-Income Countries, expanding beyond mental health to include other Non-Communicable Diseases and related risk factors.
- **Global health impact for policy and action:** Integrating Community Health Participatory interventions into crisis-response strategies presents a significant opportunity for Non-Communicable Diseases prevention and control in Low- and Middle-Income Countries, strengthening health system resilience and fostering community cohesion in crisis-affected settings.

## Background

Non-communicable diseases (NCDs) account for 74% of global deaths [1], posing a significant burden on individuals, communities, and healthcare systems [2]. This challenge is particularly concerning in low- and middle-income countries (LMICs) [3], which bear two-thirds of the global NCD burden [1]. NCDs include not only physical conditions such as cardiovascular disease, diabetes, cancers, and chronic respiratory illnesses but also mental health disorders, which are increasingly recognized as a critical part of the NCD agenda [3]. As LMICs undergo an epidemiological transition, the rising prevalence of NCDs further strains healthcare systems already contending with limited resources and infrastructure [4]. Recognizing the urgency of the situation, the Sustainable Development Goals have emphasized reducing NCD-related mortality by 2030 [5].

Effective NCD prevention and control requires a comprehensive approach that combines community-based interventions with a focused effort [6]. However, implementing these strategies remains a challenge, especially in LMICs that disproportionately experience the effects of humanitarian crises and conflicts [7]. Such crises exacerbate healthcare demands while undermining the capacity of health systems through disrupted secondary and tertiary services, inconsistent access to healthcare providers and medications, and escalating costs [8,9]. Furthermore, competing priorities in crisis-affected areas to tackle common communicable diseases often distract limited resources away from the consistent care needed for NCDs. Hence, under conditions of crisis, regular NCD care is often disordered and rarely prioritized [10].

Over the past decade, researchers have highlighted the critical role of communities as agents of change in health systems, especially within the context of LMICs [11]. Community Based participatory research (CBPR) is a collaborative methodology that establishes structures for community participation, empowering communities to address the problems being studied and utilise their resources for action [12]. Health researchers and practitioners are now encouraging the application of CBPR to tackle health disparities, particularly in managing NCDs [6,13]. These efforts are framed under community health participatory (CHP) interventions, which utilises CBPR core principles in health context. Examples of CHP in NCDs include a trial conducted in rural Bangladesh (2019) to address Type 2 Diabetes Mellitus (T2DM), which resulted in a significant reduction in the combined prevalence of T2DM and intermediate hyperglycemia [14]. Similarly, a study from Zimbabwe (2019) demonstrated improved awareness regarding hypertension, resulting in primary prevention through community empowerment and the establishment of Village Health Workers as a link between the community and healthcare delivery [15].

Despite the demonstrated potential of CHP interventions in managing NCDs, its application in crisis-affected settings remains limited and underexplored [16], with LMICs like Syria, Afghanistan, Iraq, Pakistan, Yemen and Nigeria being most affected [17]. The unique complexities of these settings necessitate a deeper understanding of how CHP interventions can be adapted and implemented effectively in crisis-affected environments. Therefore, this scoping review has examined the adoption of CHP interventions in crisis-affected LMICs to prevent and control NCDs. Additionally, it explores the inherent barriers and facilitators influencing the implementation of these interventions in contexts of crisis, including natural disasters, pandemics, conflicts, and wars. By addressing this research gap, this review aims to provide insights into the potential of CHP approaches to mitigate the NCD burden in some of the most challenging and resource-constrained environments in LMICs.

The specific research questions are presented in Table 1.

**Table 1.** Research questions.

Topic	Research questions
Intervention	What CHP interventions have been administered for the prevention and control of NCDs in crisis affected LMICs?
	What were the aims/objectives of these CHP interventions?
	Who were the target population for these interventions?
	What were the research methods used?
	What were the health and process outcomes of the interventions?
	What was the nature and extent of community participation?
Barriers and facilitators	What were the barriers to community participation in the intervention?
	What were the facilitators of community participation in the intervention?

## Methods

The scoping review was conducted using the multi-stage Arksey and O’Malley framework, incorporating refinements proposed by Levac et al. [18] and adhering to methodological guidance from the Joanna Briggs Institute (JBI) [19]. The findings of this scoping review are reported following the Preferred Reporting Items for Systematic Reviews and Meta-Analyses Extension for Scoping Reviews (PRISMA-ScR) guidelines [20]. The protocol was prospectively registered on the Open Science Framework (OSF) and is accessible online for a detailed description of the methodology [21] (registration link: https://osf.io/eu95b).

### Eligibility criteria

Primary research articles (qualitative, quantitative or mixed methods) and grey literature in English language were included if they reported on community-based interventions for NCD prevention or control in crisis-affected LMIC populations. Crisis-affected populations were defined as those experiencing or recovering from armed conflict, displacement, natural hazards, or political instability, and included internally displaced persons (IDPs) and refugees residing in LMICs. The LMIC population was defined using World Bank income classification (2024) [22], which includes three subgroups: lower income countries (LICs), lower middle income countries and upper middle income countries (UMICs). The target population was adults aged 18+ years, though studies with mixed-age populations were eligible if subsets met the criteria. Eligible outcomes were any outcomes related to NCD prevention or control at the individual, community, or health system level. Excluded were non-experimental studies, review articles or protocol papers and studies with a different focus (such as those addressing sexual violence etc.). Additionally, narrative reports and conference abstracts were excluded that lacked original data or did not meet our eligibility criteria. However, qualitative or other non-experimental papers – including field reports – were included if they reported findings from experimental interventions and provided relevant implementation insights aligned with our study objectives. The detailed operational definitions are provided in supplementary Table S1.

### Databases and search strategy

The search strategy was developed based on the research objectives and focused on four broad areas including CHP, NCDs, conflict, and LMIC. It was implemented in three stages: an initial search on PubMed and Google Scholar, followed by applying the final strategy across PubMed, Web of Science, Scopus, and Google Scholar. However, for Google Scholar, only the first 300 results were included in the screening phase, following the recommendations of Haddaway NR et al. for conducting systematic reviews [23]. Targeted exploration of relevant reports, reviews and articles’ reference lists was also done and retrieved manually. The databases were last searched on 30 January 2024 and no publication date limits were applied. Full applied search strings can be found in supplementary Table S2.

### Study selection

The results were imported into Covidence; a robust web-based software to manage reviews [24], using the Endnote library. The duplicate entries were automatically identified and managed, ensuring the integrity of the review process. A team of 15 reviewers, priorly trained in Covidence and scoping review (SI, NK, AT, NS, MA, RZ, FK, AK, ZK, FH, ShA, NA, AsK, ARS, HE), evaluated the eligibility of all titles and abstracts, followed by a full-text review of all potentially eligible studies. This approach yielded a high level of agreement among reviewers, with 90% consensus on both the title and abstract, and the full-text screening, as each study was reviewed by two reviewers independently. The screening phase was iterative, with additional articles manually added after reviewing reference lists of relevant studies. Any conflicts or unresolved disagreements that arose during the screening process were resolved by two independent reviewers (SA and SI). SA is an Associate Professor Global Public Health with experience in systematic reviews and NCD research, while SI, an early career researcher, was the lead researcher for this study and brought detailed contextual and methodological knowledge.

### Data extraction

The data was extracted by creating a structured data extraction sheet on Covidence and tested by piloting multiple studies. A data extraction guidance sheet was made that included definitions and extraction categories for the ease and understanding of reviewers. Any discrepancies in the extracted information were resolved through discussion or re-examination of the articles as needed.

The extraction categories included: study characteristics, aim of the study, study design, setting and target population, type and characteristics of intervention, key findings, process outcomes, and barriers to community participation in the intervention and facilitators to community participation in the intervention. Additionally, the nature and level of community involvement in the intervention was assessed using Kirk Wallace Spectrum of involvement [25], while the geographical urban and rural area was identified based on descriptions provided in the original studies.

### Data analysis

A narrative analysis of the extracted data was conducted based on the pre-identified categories aligned with the research questions and cross-checked to ensure accuracy of the data. Descriptive summaries were generated to quantify study types, settings, themes and types of intervention. The barriers and facilitators to intervention were identified, coded and systematically grouped to identify overarching qualitative themes using thematic analysis. Additionally, the analysis was cross- referenced with the PRISMA checklist, ensuring that the synthesis and presentation of results aligned with the objective and research questions.

## Results

**Figure 1.**
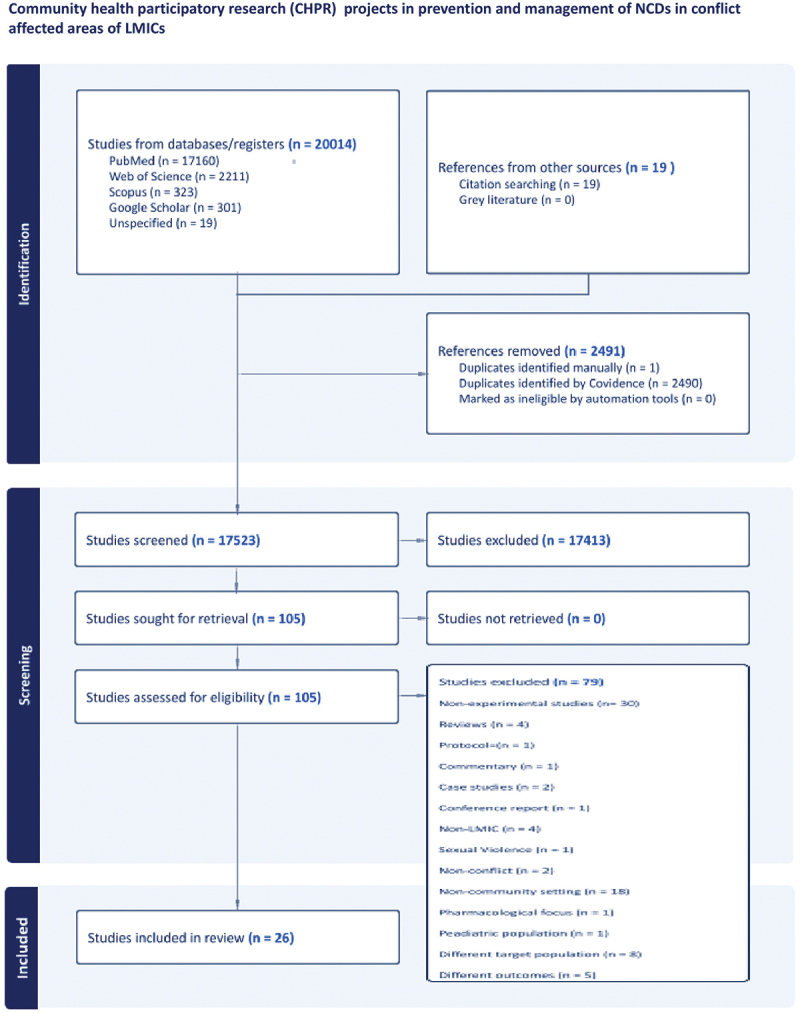
PRISMA flowchart.

The search strategy yielded 20,014 results, with 2491 duplicates removed. After screening 17,523 titles and abstracts, 105 articles were selected for full-text review. While, the remaining 17,418 were excluded as they did not meet one or more of the pre-specified eligibility criteria. In the second phase of screening for full-text review, 79 studies out of 105 were excluded for not meeting the inclusion criteria, resulting in 26 studies being included for data extraction (Refer to Figure 1: PRISMA flowchart).

### Geographic profile of the studies

**Figure 2.**
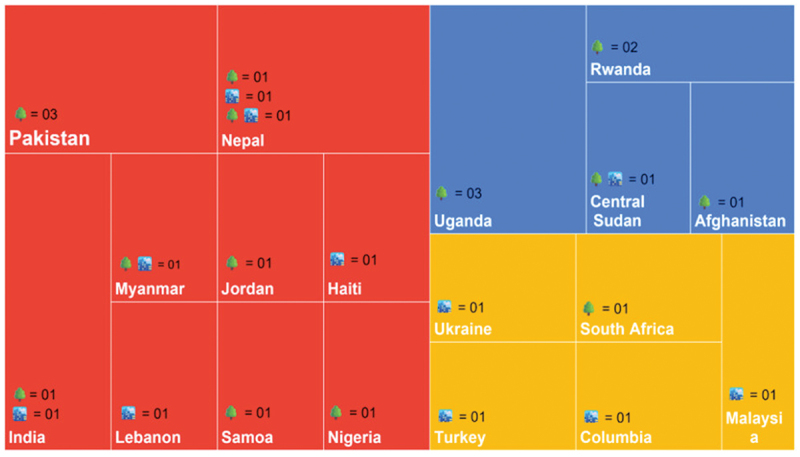
Regional and geographical distribution of studies.

Of the 26 included studies, the majority (*n* = 21) were published from 2017 onwards, with a peak in 2021 (*n* = 04; [26–29]) followed by 2022 (*n* = 03; [30–32]) and 2023 (*n* = 03; [33–35]). Most studies (*n* = 14) were conducted in Lower middle income countries [26,28,30,35–45], followed by LICs (*n* = 07), and UMICs (*n* = 05). Uganda [29,31,33], Nepal [28,39,40], and Pakistan [36,43,44] were the most frequently represented countries, with three studies each. Other countries included India (*n* = 2), Rwanda (*n* = 2), Myanmar, Jordan, Haiti, Lebanon, Samoa, Nigeria, Sudan (Central), Afghanistan, Ukraine, South Africa, Turkey, Colombia, and Malaysia. CHP interventions were predominantly implemented in rural settings (*n* = 14; [26,29,31–33,35,36,39,42–47]), eight studies focused on urban settings [27,34,37,38,40,41,48,49] and four studies encompassed both urban and rural contexts [28,30,50,51]. Notably, studies from UMICs predominantly focused on urban settings, those from LICs largely addressed rural contexts, while studies from Lower middle income countries covered both urban and rural areas as illustrated in Figure 2.

### Study designs and context

Randomised controlled trials (RCTs) were the most common study design (*n* = 16), comprising ten definitive trials [27–29,33,41,44,45,47–49], five feasibility studies [26,36,39,42,43], and one pilot study [34]. Four of these RCTs employed a mixed-methods approach [34,36,39,43]. Additionally, eight studies used quasi-experimental designs [31,32,35,38,40,46,50,51], one employed an experimental post-intervention design [30], and one was a field report that documented the expansion of a volunteer community outreach program for Syrian refugees between 2016 and 2017 [37].

The studies addressed various crisis contexts, including military armed conflicts (*n* = 08 [27,33,36,42–44,46,47]), humanitarian crisis (*n* = 07; [28–31,34,35,51]), natural disasters (*n* = 06; [38–41,45,48]), and civil wars (*n* = 05; [26,31,37,49,51]). The studies were predominantly funded by international bodies (*n* = 04; [40–42,44]), with significant contributions from the United States (*n* = 09; [27,28,30,33,34,36–39]), the United Kingdom (*n* = 03; [26,32,35]), the Netherlands (*n* =  = 02; [46,50]), Canada (*n* = 02; [29,47]), and Norway (*n* = 01; [51]). Additionally, two studies reported national funding, one each from the governments of Malaysia [48] and Uganda [31]. While three studies did not report any funding [43,45,49].

### Target population and focus

The 26 included studies targeted diverse populations, including vulnerable populations such as refugees and displaced persons (*n* = 8), adults affected by conflict, violence, or disasters (*n* = 7), women (*n* = 5), adults with mental health issues or psychological distress (*n* = 3), community members and families (*n* = 6), and veterans (*n* = 1) (details provided in supplementary Table S3). The majority of interventions focused on mental health issues, particularly depression and anxiety, while also incorporating intersectoral approaches such as social cohesion (*n* = 01; [50]), disaster preparedness (*n* = 02; [40,41]) and maternal health (*n* = 02; [31,43]). Additionally, one study addressed non-communicable diseases (NCDs) broadly [37], while one specifically targeted cardiovascular disease (CVD) [32], breast cancer [49], and alcohol [31] respectively. The overarching theme was prevention (*n* = 11; [26,32,33,38–43,46,49,51]) and control (*n* = 07; [27–30,36,37]), with eight studies covering both aspects (*n* = 08; [31,34,35,44,45,47,48,50]).

### Intervention characteristics

Across the included studies (*n* = 26), some interventions were based on pre-existing programs, notably Group Problem Management Plus (PM+) (*n* = 04; [26,36,39,44]), Psychological First Aid (PFA) (*n* = 01; [29]), Suicide Prevention (CASP) (*n* = 01; [42]) and WHO’s Self-Help Plus (SH+) (*n* = 01; [33]). In contrast, a number of interventions were newly developed or context-specific, such as Alcohol Use Communication Intervention [31], Asiasiga: a Samoan intervention [45], Breast & Cervical Cancer Education [49], and Community-Based NCD Care [37]. The intervention details are summarised in Table 2.

**Table 2.** Intervention characteristics.

Author (Year of Publication)	Name of Intervention	Key Components	Setting	Materials	Duration & Frequency	Delivered By	Mode
Akhtar et al. (2021) [26]	Scalable Transdiagnostic Psychological Intervention (PM+)	Stress management, social support strengthening	Refugee camps	Manual	5 weeks (120 min sessions)	Non-specialist facilitators	Face-to-face, group (6–12)
Bogdanov (2021) [27]	Community-Based Transdiagnostic Psychotherapy	Psychoeducation, safety, substance use reduction, coping	Social services, NGOs	CETA manual	5–12 weeks	Community Providers	Digital & private sessions
JordansMJD et al. (2021) [28]	Group Problem Management Plus	Problem-solving, stress management, behavioral activation, and social support.	Community centers, and civil society organization offices	Manual by WHO	5 weekly 2.5 hours session	Non- specialist facilitators	Face to face, group
Musisi et al. (2021) [29]	Psychological First Aid (WHO)	Emotional support, needs assessment, mobilizing referrals	Health centers, community hubs, parks, places of worship	WHO-PFA materials	One day/as needed	Volunteer Village Health Teams	Face-to-face
Lee et al. (2022) [30]	Psychosocial Support Focal Point Response	Psychoeducation on stress, coping, and cognitive restructuring skills.	Residential	Handouts, audio recording, telephone services	5 Months	CETA community counselors	Face to face group
Agiresaasi et al. (2022) [31]	Alcohol Use Communication Intervention	Messages on alcohol risks, myths, and cessation	Residential, village, church meetings	Visual images, translated messages	Not specified	CHWs, Village Health Teams	Face-to-face, house-to-house
Mphekgwana et al. (2022) [32]	Community Action Model for NCDs	Health education (especially regarding CVD), motivational interviewing	Residential	Pamphlets	3 months, follow-up	CHWs	Face-to-face, group
Augustinavicius et al. (2023) [31]	Self-Help Plus (SH+)	Stress management, acceptance therapy	Refugee camps	Audio course, self-help book	5 weekly 2-hour sessions	Lay Facilitators	Face-to-face, group
Rattner et al. (2023) [34]	Community-Based Psychosocial Support (CB-PSS)	Problem-solving, expressive cultural activities	Virtual & community centers	Not mentioned	8 weekly 2-hour sessions	Psychosocial Agents	Hybrid (group/individual)
Paphitis et al. (2023) [35]	Counseling on Wheels for Mental Health & Peacebuilding	Psychoeducation, relaxation, resilience building	Not specified	Flexible toolbox	3 weekly sessions (1 hour)	Semi-skilled Counselors	Face-to-face, group (8–10)
Khan et al. (2017) [36]	WHO trans-diagnostic intervention – Group PM+	Psychoeducation, problem solving, stress management and self-care strategies	Residential areas	Manual	Five weekly sessions (2 hours)	Local female lay helpers	Face to Face, group
Sethi et al. (2017) [37]	Community-Based NCD Care	Health promotion, disease control	Refugee settlements	Flip charts, posters, checklists, WhatsApp	2 months	Refugee Outreach Volunteers	Face-to-face, group
Becker et al. (2009) [38]	Psychosocial Care Initiative	Crisis intervention, self-care, caregiving skills	Not specified	Manuals, therapeutic activities	3 months	CHWs	Face-to-face, group (10 women)
Sangraula (2020) [39]	WHO Group Problem Management Plus (PM+)	Stress management, problem-solving, behavioral activation	Not specified	WHO-based materials	5 weekly sessions (2.5–3 hours)	Gender-matched facilitators	Face-to-face, group (6–8)
Welton-Mitchell et al. (2018) [40]	Mental Health & Disaster Preparedness	Coping strategies, disaster response training	Not specified	Adapted manual, disaster supply kit	3 days	Nepali Facilitators	Face-to-face, group (20)
James et al. (2020) [41]	Mental health and disaster preparedness intervention	Facilitated discussions, peer support, safety practices, coping skills, and hands-on disaster preparedness training.	Not specified	Standardised Manual	3 days	Trained Haitian lay mental health workers	Face-to-face, group (20)
Vijayakumar et al. (2017) [42]	Suicide Prevention (CASP)	Emotional support, coping skills	Refugee camps	Safety Planning Cards	15 months (bi-monthly visits)	Community Volunteers	Face-to-face, individual
Khan et al. (2017) [43]	Local Psychoeducation Intervention	Listening skills, social support, stress management	Residential	Manual, counseling cards	2 sessions (20 min & 1 hour)	CHWs	Face-to-face
Rahman (2019) [44]	A brief psychological intervention- Group PM+	Psychoeducation, goal setting, motivational interviewing, stress management, behavioral activation, and social support.	Space in houses provided by the LHW.	Locally relevant visuals and case-based narratives.	Five weekly group sessions (2 hours)	Local facilitators	Face to Face group (68)
Tamasese et al. (2020) [45]	Asiasiga: a Samoan intervention to address the immediate mental health needs	Instilling hope, tsunami survivor stories, narrative therapy, and arrangement of material support.	Household visits	None	3 weeks to 3 months	Samoan Pastoral workers	Face-to-face, house-to-house (Family based)
Scholte et al. (2011) [46]	Psychosocial Sociotherapy	Problem-solving, mental health improvement	Not specified	Training manual	15 weeks, 3-hour weekly sessions	Group Leaders	Face-to-face, group (10–15)
Khoja (2016) [47]	Conventional & Telehealth Mental Health Solutions	SMS mental health messages, stigma reduction	Community Town Halls	Mobile app, SMS messages	150 town hall meetings, 11,500 texts	CHWs	Digital & group
Krishnaswamy et al. (2012) [48]	Early Mental Health Intervention	Psychoeducation, PTSD symptom recognition, stress management	Residential	WHO handbook-based materials	1-day module	Trained Volunteers	Face-to-face, family-based group
Erenoğlu et al. (2020) [49]	Breast & Cervical Cancer Education	Disease awareness, self-exam training	Public Education Centers	Brochures, videos, presentations	1 day (1–2 hours)	Researchers, Translators	Face-to-face, group (7–10 women)
Verduin et al. (2014) [50]	Sociotherapy for Social Capital	Trust-building, coping strategies, group exercises	Informal settings	Sociotherapy training materials	15 weekly meetings	Group Leaders	Face-to-face, group
Sanhori (2019) [51]	Mental Health Stigma Awareness	Information on mental illness, stigma reduction	Not specified	Brochures, posters	5 sections (4 hours each)	Mental Health Professionals	Group-based intervention

About 15 studies incorporated contextual adaptations [26–28,33,34,36–41,43,44,47,50], while others directly applied standardized models (*n* = 11; [29–32,35,42,45,46,48,49,51]). The majority of studies (*n* = 23) utilised group-based interventions, primarily in-person, with sessions conducted weekly over 3 to 15 weeks (*n* = 19; [26,28–39,44–46,48–50]). Some involved single sessions (*n* = 04; [43,48,49,51]), multi-day workshops (*n* = 02; [40,41]), or bi-monthly meetings over 15 months (*n* = 01; [42]). In most studies, trained community members (e.g. health workers, volunteers, pastors, and group leaders) facilitated sessions.

Common strategies included collaborative problem-solving, group discussions, role-playing, strengthening social support, group prayers, stress management, goal setting, motivational interviewing, and health education. Many interventions also encouraged behavioural changes such as increasing physical activity, improving diet, quitting smoking, and practicing relaxation techniques. Sessions were delivered in community-based settings, including homes, informal venues, refugee camps, community centers, parks, places of worship, and NGO facilities.

A few studies used hybrid or individual approaches (*n* = 04; [27,34,42,47]). One combined virtual component with in-person sessions [47], while three implemented individual sessions [27,34,42]. Individual sessions were conducted in refugee camps [42] and private rooms [27]. While, one study adopted a hybrid model due to COVID-19 [34]. Individualized interventions focused on behavioral activation, psychoeducation [27], emotional support, coping strategies [42], and culturally relevant expressive activities [34].

Community mobilisation strategies were frequently employed to enhance engagement and sustainability, involving local leaders (*n* = 04; [35,41,45,50]), religious figures (*n* = 02; [45,50]), NGOs (*n* = 04; [35,37,38,50]), and family members (*n* = 02; [36,45]). A study also integrated peacebuilding activities [35], including forums, educational partnerships, and community murals. Capacity-building efforts trained lay facilitators (*n* = 08; [29,30,33,36–38,40,41]), such as Community Health Workers (CHWs) and Refugee Outreach Volunteers (ROVs), ensuring sustainable intervention delivery.

### Reported outcomes and process outcomes

The studies assessed various indicators based on their objectives, with some focusing on feasibility rather than full-scale evaluation. Twenty-five of the studies documented improved results post-intervention, demonstrating reductions in distress, anxiety, depression, and post-traumatic stress, along with improved overall wellbeing and raised awareness for breast cancer and NCD risk factors [26–50]. The remaining one study reported no effect of the community awareness intervention on mental health stigma reduction [51]. It is important to note that all reported outcomes and process outcomes are based on authors-reported findings, and the quality of the studies or their reported outcomes was not formally assessed.

Several studies reported strong retention rates, participant comfort, and satisfaction among individuals attending at least one to three intervention sessions, particularly in PM+ interventions [26,36,39,44]. Two studies explored the feasibility component as a successful outcome of the CB-PSS group intervention and locally developed psycho-educational intervention [34,43]. The additional positive impacts of the interventions included noticeable civic participation [50], increased social cohesion (*n* = 03; [40,41,50]) and women’s increased trust in LHWs [43]. However, the outcomes varied when the mode of delivery shifted from in-person to hybrid and remote modalities [34].

Studies assessing adherence levels showed moderate-to-high levels across facilitators [43] and participants [42], healthcare providers, young adults and the public [27]. One study emphasized the success of an outreach strategy, noting that 93% of participants received handouts, and over 85% engaged with the materials [30], with 29% of respondents indicating positive role of handouts in daily routine and 65% reported occasional use. Reach to participants was improved through using different reading materials [30], the use of mobile applications [37], and CHW’s involvement [36]. Further details are mentioned in supplementary Table S4

### Level of community participation within the interventions

**Figure 3.**
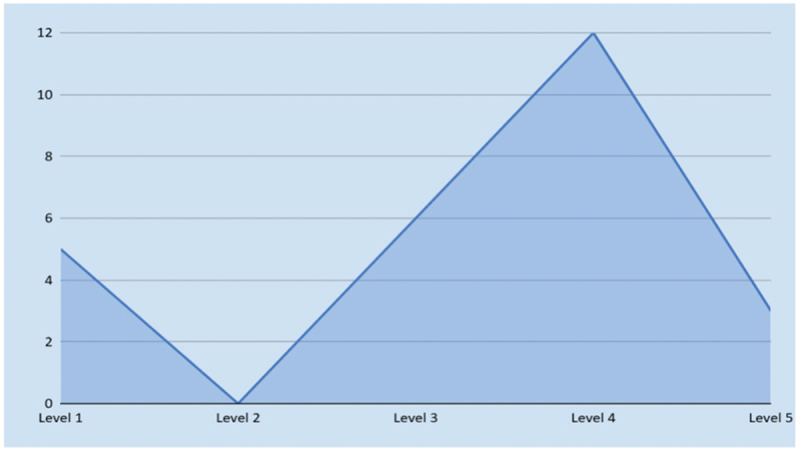
Levels of community participation and engagement.

The review identified varying levels of participant engagement in interventions as shown in Figure 3.

A total of 12 studies adopting a collaborative approach (Level 4) [26–28,34,35,38–41,43,44,48]. These interventions involved shared decision-making and problem-solving alongside facilitators, emphasizing joint responsibilities. Examples included structured peer-support groups, experiential activities, and disaster preparedness training.

Six studies demonstrated the active involvement of participants (Level 3) [29,30,33,36,37,42], focusing on their role in delivering interventions, such as Village Health Teams (VHTs) and Refugee Outreach Volunteers (ROVs) providing Psychological First Aid (PFA) or behavior change sessions. These approaches tailored interventions to community needs but did not grant full decision-making authority.

A smaller number of studies (*n* = 05) reported informing participants (Level 1) [31,32,47,49,51], primarily through raising awareness via town hall meetings, digital messages, and sessions on topics like mental health, cardiovascular disease (CVD), and alcohol use during pregnancy.

The highest level of engagement, empowerment (Level 5) [45,46,50], was less frequently observed (*n* = 03). These interventions granted participants or community leaders full control over decision-making and implementation. Examples included Samoan pastoral workers integrating cultural practices into family engagement and community leaders adapting interventions using trust-building, spirituality, and role-plays tailored to group characteristics (Refer to Table 3).

**Table 3.** Nature and extent of community involvement in the intervention.

Study	Description
**Level 1 – Informing**: Participants are informed about their health and the intervention. They receive information but have no direct influence on decision-making.	
Agiresaasi [31], Mphekgwana [32], Khoja [47], Erenoğlu [49], Sanhori [51]	Screening for risk factors and specific NCDs, along with counseling and awareness initiatives. Additionally, raising awareness through stigma reduction specifically for mental illness.
**Level 2- Consulting**: Participants are asked for their opinions and feedback on health interventions. Their input is considered, but the final decision is made by the organizers/researchers.	
—-No study classified in level 2—–	
**Level 3 – Involving**: Participants are involved in the design and implementation of the intervention. Their input directly influences the decisions made, but they do not have decision making authority.	
Musisi [29]	Village Health Teams (VHTs) were trained to deliver Psychological First Aid (PFA) to trauma survivors, with the community actively identifying victims, providing PFA, and offering ongoing support under professional supervision.
Lee [30]	Utilizing local resources in implementing and tailoring the approach to meet community needs, fostering a sense of participation in the intervention process
Augustinavicius [31]	During SH+ sessions, facilitators turned the audio recording on and off, answering questions, directing individual exercises and small group discussions in a highly scripted manner and ensuring participant safety (eg, responding to any risk- related issues).
Khan [36]	LHWs delivered the intervention to psychologically distressed pregnant women and their families at home, with active participation in structured sessions.
Sethi [37]	Facilitated behavior change sessions for refugees, trains ROVs on key health topics, and supports them as community health links, with CHPs conducting monthly participatory education sessions.
Vijayakumar [42]	Meetings conducted with high-risk individuals and involved in drafting list of possible warning signs, possible strategies for suicide prevention, and a list of possible support in the community to reduce depression and suicide
**Level 4 – Collaborating**: Participants work in partnership with the organisers/researchers throughout the entire process, sharing partial power and responsibility for decision-making and implementation.	
Akhtar [26], JordansMJD [28], Sangraula [39], and Khan [43]	Active community participation in identifying problems and setting goals for their mental health with the help of semi-skilled facilitators
Rattner [34], Rahman [44], Krishnaswamy [48]	Trained facilitators repeatedly engaged the community decision-making and problem-solving, emotional expression of anguish and grief for as long as the family felt it necessary
Bogdanov [27], Paphitis [35], Becker [38], Welton-Mitchell [40], James [41]	Participants actively engaged through facilitated discussions, personal experience sharing, peer support exchange, and coping skills practice, with structured sessions led by a standardized manual and external counselors, reflecting a partnership approach.
**Level 5 – Empowerment**: Participants have full control over the decision-making process and implementation of the intervention. They are given the resources and authority to make and implement decisions independently.	
Tamasese [45]	The active participation of Samoan pastoral workers, who engaged with families within their cultural context, provided spiritual and emotional support while assessing broader needs. This approach fostered a supportive environment that promoted community resilience and well-being, aligning with local practices and values.
Scholte [46], Verduin [50]	As community leaders were trained, there was no specific protocol, only principles and phases. Group leaders were allowed to adapt their routines based on the characteristics of their groups (e.g. trust levels, nature of problems) and their own affinity and experience, adjusting the emphasis on elements like rules, role plays, and spirituality.

### Barriers and facilitators to CHP interventions

**Figure 4.**
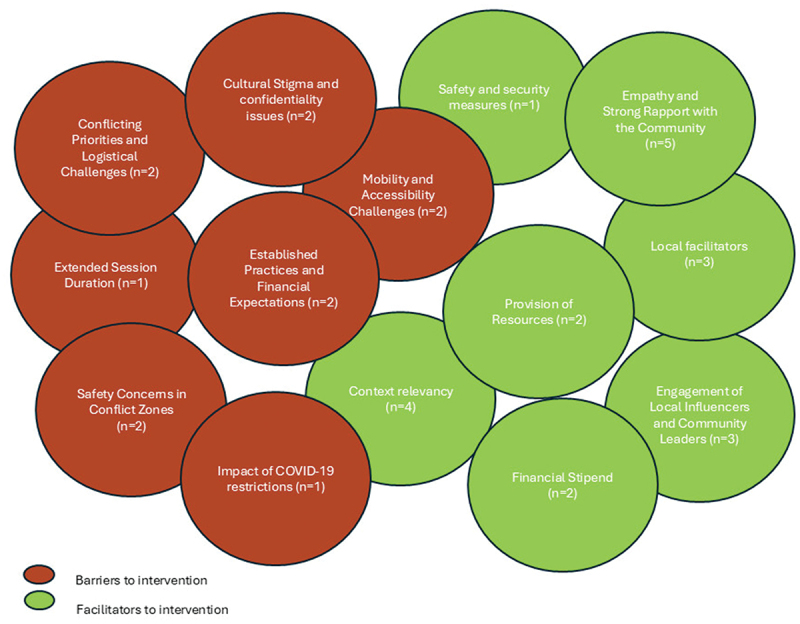
Barriers and Facilitators to intervention.

Of the 26 included studies, the majority (*n* = 17) did not report on the barriers and facilitators associated with CHP interventions. Just one study explicitly identified exploring barriers and facilitators as an objective [34]. Four studies provided details on barriers and facilitators to CHP in their results sections [34,36,39,42], and an additional five studies discussed potential barriers and facilitators within their discussion sections [26,27,44–46]- as shown in Figure 4.

### Barriers to intervention

#### Competing commitments and logistical issues

Two studies reported that competing activities and busy schedules of community members significantly affected participation in the interventions [26,34]. Participants frequently missed intervention sessions due to conflicting commitments, including appointments related to residency and refugee status, mobility challenges arising from ongoing conflicts, additional responsibilities and barriers stemming from lockdown measures, childcare, family obligations, and other personal engagements (*n* = 03; [26,27,34]). Furthermore, two studies (*n* = 2) noted that financial constraints [27,34], such as the costs associated with food and transportation, hindered participants’ ability to attend sessions. One study [34] that was delivered online during COVID-19 reported technological barriers including disruption in internet connection or availability of gadgets.

#### Established practices and financial expectations

Two studies noted that the lack of monetary incentives for participation hindered retention in the intervention [36,39]. One study highlighted that previous programmes implemented by international organisations in these areas regularly provided incentives, creating an expectation among participants for monetary benefits [36]. This expectation made it challenging to motivate participation in the current intervention.

#### Confidentiality and cultural stigma

Two studies reported that due to the mental health focus of the intervention some participants hesitated to share their problems in group settings and preferred to discuss them individually with facilitators, especially when family members were involved in the same group [36,39]. Additionally, one study documented reluctance among male participants to engage in the intervention due to cultural stigma surrounding male participation and the tendency to keep personal issues private [37].

#### Security and safety concerns

Two studies [34,39] highlighted security and safety concerns, particularly during the COVID-19 pandemic and ongoing conflicts. These issues contributed to disrupted attendance and adversely affected in-person participation in the interventions.

#### Duration of the intervention

One study indicated that the longer duration of intervention sessions posed an obstacle to participation both during the intervention period and within individual sessions [36]. Conversely, another study [27] reported that shorter or briefer intervention durations might also have negatively impacted participation, particularly given the ongoing conflicts that necessitated more mental health sessions.

### Facilitators to intervention

#### Local facilitators and context relevancy

Several studies reflected upon the positive role of lay facilitators for the intervention that has provided a community face to the intervention and encouraged community buy-in for the program (*n* = 04; [36,39,42,45]). Furthermore, training of the facilitators for the cultural relevance of the invention aided the smooth progress during the intervention (*n* = 03; [39,42,45]). The use of local cultural practices like ‘Asiasiga’ enhanced the acceptability of interventions, ensuring that they were well received by the community [45]. Employing local idioms for distress in a group format helped reduce self-stigma and increase social support [39].

#### Empathy and strong rapport with the community

Facilitators who were culturally sensitive and had built a good rapport and empathetic relationship with the community members fostered a supportive environment, encouraging participants’ engagement and retention (*n* = 04; [34,36,39,42]). The participants therefore felt more comfortable discussing their problems with them and this resulted in active participation during the intervention. Group settings that promoted equality and offered a platform to unburden emotional distress were effective in building trust and facilitating open communication and also facilitated people engaging in the intervention (*n* = 03; [39,42,44]).

#### Provision of resources and financial stipends

To enhance accessibility within the community, two studies reported that providing resources and financial support helped overcome barriers to participation [34,36]. This included providing resources like transport stipends, mobile data, and flexible scheduling.

#### Engagement of local influencers and community leaders

Two studies reported that engaging community representatives and community peer advocates fostered trust and facilitated intervention sessions and ongoing follow-up with participants [34,36]. While, one study reported that involvement of local actors during the intervention and mobilisation positively impacted the intervention delivery [34]. Furthermore, collaborations with local churches, village groups, and community leaders strengthened participation and built trust within the community. These partnerships were crucial for successful implementation and sustainability of the interventions [34,45].

#### Safety and security measures

One study reported that implementing safety measures was essential for sustaining participants’ engagement and retention, particularly in conflict-affected settings where security concerns are prevalent [34]. These measures included ensuring safety during intervention sessions, disseminating critical information about security conditions, and arranging safe transportation for participants.

## Discussion

This scoping review provided an overview of the characteristics, implementation strategies, community participation, and outcomes of CHP interventions targeting NCDs in crisis-affected LMICs. Notably, five key insights have emerged: (1) the dominance of mental health interventions among NCD-related CHP studies; (2) the geographical and economic concentration of studies in LMICs, particularly rural settings; (3) methodological diversity with a predominance of randomised controlled trials (RCTs); (4) variation in levels of community participation; and (5) underreporting of barriers and facilitators despite their critical influence on intervention delivery.

These findings underscore the growing body of evidence supporting CHP interventions as a vital strategy for NCD prevention and control in these challenging contexts that emphasize community engagement and promote inclusivity [52]. Notably, the global focus has recently shifted to an all-hazard approach, integrating NCD care within humanitarian responses [53]. This trend is reflected in our findings, with most studies published after 2017 – potentially following WHO’s recognition in 2016 of the significant impact of emergencies on individuals with NCDs [54].

### Mental health dominance within NCD-focused interventions

Mental health emerged as the most frequently studied area for CHP interventions (*n* = 22) in crisis-affected LMICs, specifically depression, anxiety, and psychosocial distress. This reflects a global trend in humanitarian and health research, where mental health and psychosocial support (MHPSS) is increasingly prioritised as a core component of crisis response, as reflected in the WHO’s *Mental Health Gap Action Programme* and the Inter-Agency Standing Committee (IASC) Guidelines on MHPSS in Emergency Settings [55,56]. In contrast, other NCDs received limited attention. This prioritisation may be driven by the visibility of psychological distress, its strong association with trauma, and the availability of scalable interventions such as Group Problem Management Plus (PM+), Self-Help Plus (SH+), and culturally adapted psychosocial models deliverable by trained lay personnel in low-resource contexts [57,58]. Furthermore, the persistent psychological toll of the crisis further contributes to the prioritisation of mental health, as these challenges extend well beyond the resolution of physical injuries [59].

Mental disorders are among the leading causes of disability worldwide, and conflict-affected populations experience substantially higher prevalence rates than the global average – recent WHO estimates suggest that approximately 22.1% of individuals in such settings have a mental disorder at any given time, with 5.1% experiencing severe conditions such as psychosis, severe depression, or severe anxiety disorders [60]. By contrast, other NCDs, although accounting for 85% of premature NCD mortality occurring in LMICs [61], progress gradually and often lack immediate recognition despite their long-term impact. However, this side of the NCD-focused intervention research in humanitarian settings remains sparse, contributing to what has been termed the ‘invisible burden’ of chronic disease in crises [62]. This review quantifies the imbalance between mental and physical health, highlighting the need for integrated approaches that address both NCD domains.

### Geographic and socioeconomic concentration of studies

Most studies were conducted in lower middle income countries (*n* = 14), particularly in South Asia and Sub-Saharan Africa. A majority took place in rural areas, although a few explored urban or mixed settings. This distribution reflects both the frequency and visibility of crises in these regions.

LICs and Lower middle income countries are more vulnerable to both conflict and disaster, and often lack resilient health infrastructure [54,63]. Conversely, crises in UMICs – often urban, politically sensitive, and less reliant on international aid – may be underreported or under-researched. This review reinforces the need to expand CHP research into underrepresented contexts, especially urban conflict zones in UMICs.

### Variation in study designs

Sixteen of the included studies were RCTs, supplemented by quasi-experimental and mixed-method designs. This suggests increasing rigour in evaluating community-based interventions in humanitarian settings. Mixed-method studies offered valuable insights into acceptability, feasibility, and cultural fit, supporting earlier findings that advocate for context-sensitive methodologies in complex emergencies [63,64]. However, the scarcity of longitudinal data and sustainability metrics suggests an ongoing gap in implementation science within crisis settings [65].

### Community involvement and target populations

Crisis disrupts healthcare systems, and limited resources make it challenging to implement and sustain NCD interventions that require continuous care and medication adherence [66]. In such settings, community involvement fosters a sense of ownership and empowerment, enhancing the effectiveness of interventions [67]. This was also reflected in our findings, where interventions that engaged lay actors, community groups, or local leaders tended to demonstrate higher levels of acceptability and adherence compared with those relying solely on professional delivery models [68]. Our findings add to this evidence by showing that the degree of community involvement and the vulnerability of target populations directly shaped intervention.

While this review included studies involving general adult community members, some explicitly targeted vulnerable populations, such as refugees, women and internally displaced people. These studies highlighted the added complexity of displacement, stigma, and competing livelihood demands, which often limited intervention reach and sustainability. This also aligns with broader literature, which highlights that crises disproportionately affect LMIC populations and often have extended consequences for neighboring countries that host displaced individuals [69].

Additionally, many studies emphasized cultural adaptation, ensuring interventions were relevant and sensitive to local norms and practices – an essential factor for their acceptability and effectiveness [70]. Interventions that combined community ownership with tailored support for displaced or marginalized groups were better able to navigate structural constraints, illustrating the importance of contextual adaptation in humanitarian NCD responses.

### Community participation: levels and implications

The review documented a wide spectrum of community participation across studies, ranging from passive information-sharing (Level 1) to full empowerment (Level 5), implementing diverse strategies at individual, group, and community levels. Most interventions employed a collaborative approach (Level 4), promoting shared decision-making between external facilitators and community members. Only three studies seem to have achieved empowered participation (Level 5), where community actors took full ownership of intervention delivery. No study reported Level 2 participation.

When comparing participation levels, interventions granting less decision-making authority (Levels 1–3) were typically characterized by top-down information delivery, limited adaptation, and weaker sustainability mechanisms. These models were more common in short-term health education or awareness-raising initiatives and often struggled with community ownership, resulting in lower engagement and higher attrition.

By contrast, interventions with higher levels of decision-making authority (Levels 4–5) integrated cultural tailoring, relied on strong local leadership, and demonstrated greater sustainability. Empowered participation (Level 5) interventions, though rare, often embedded co-design, capacity-building of lay actors, and flexible delivery models, which fostered long-term community ownership and resilience.

These findings corroborate frameworks such as Arnstein’s Ladder of Participation and WHO’s guidance on community engagement in humanitarian settings [71,72]. This stratification highlights a persistent gap in empowerment-based models, suggesting that future interventions must prioritize genuine co-design and local leadership to ensure relevance, uptake, and sustainability [73].

### Barriers and facilitators: underreporting and implications

A major limitation of nearly all the reviewed studies was the lack of comprehensive reporting on barriers and facilitators. Only 9 of the 26 studies (>34%) reported barriers and facilitators, and just one listed it as a study objective. Common barriers included competing commitments, financial constraints, gendered stigma, technological limitations, and safety concerns. The lack of resources such as transport and mobile data further limited accessibility, while longer intervention durations sometimes led to participant fatigue. These challenges are consistent with findings from other studies, which report that high caregiving demands, displacement, and safety concerns limit the effectiveness of interventions in humanitarian settings. Additionally, failing to adjust session schedules to participants’ daily routines often leads to high attrition, further complicating implementation [74–76].

Conversely, key facilitators of success included the use of culturally sensitive approaches, engagement with local leaders, and the involvement of lay facilitators who fostered trust and support within communities [34,36,39,42,45]. Providing logistical support, such as flexible scheduling and transport stipends, also played a crucial role in reducing barriers [34,36] depicting the role of both structural enablers and trust-based relationships [77–79]. These findings align with evidence from other crisis and disaster settings, where contextually adapted interventions, local stakeholder involvement, and secure, accessible venues have been shown to enhance acceptability, sustainability, and participant retention [80–83].

The limited reporting of these factors reduces opportunities for learning and replication. By mapping these across included studies, this review emphasizes the need to embed process evaluation frameworks within CHP research to better capture implementation dynamics [64]. Taken together, our findings underscore that logistical and contextual support is not an ancillary feature but a prerequisite for delivering effective and equitable NCD interventions in crisis-affected LMICs.

### Feasibility, sustainability, and future research

Despite the promising role of CHP interventions, evidence on their feasibility and sustainability remains limited [70]. While some studies assessed feasibility, many focused on short-term outcomes, lacking comprehensive data on long-term impact and scalability. Existing evidence suggests that collaborations enhance participation and mutual learning, but reliance on external actors may limit sustainability.

Our review indicates that ownership of CHP interventions in highly mobile populations can be promoted through strategies that ensure flexibility, portability, and continuity of community roles. For example, training lay facilitators from within displaced or refugee groups enables skills and knowledge to move with communities, reducing dependence on external actors. Similarly, modular program designs – where content can be delivered in shorter cycles or adapted to changing settlement conditions – help maintain relevance despite mobility. Building partnerships with local organizations and host community structures further supports continuity by embedding interventions within existing networks rather than temporary relief operations.

Therefore, adaptability, cultural alignment of interventions and long-term ownership, that is core principle of CHP intervention, is particularly important in diverse or resource-constrained settings [28,40]. To further strengthen the evidence, expanding research to include longitudinal studies and underrepresented crisis contexts will be essential for generating actionable insights and facilitating broader implementation.

## Conclusion

This review demonstrates that community health participation (CHP) interventions can play a critical role in addressing NCDs, particularly mental health, in crisis-affected LMICs, where formal health infrastructure is often disrupted. The findings underscore an urgent need to scale up context-sensitive NCD interventions in such settings, aligning with broader calls to integrate mental health and NCD services into humanitarian response frameworks, thereby bridging the gap between communities and healthcare systems.

Levels of community participation varied widely, with most interventions adopting collaborative models but very few achieving full empowerment, reflecting a persistent gap between theory and practice. Evidence from this review suggests that interventions granting communities greater decision-making authority are more sustainable, culturally appropriate, and better aligned with local needs, yet such models remain underutilized.

Barriers such as resource shortages, displacement, and dependence on external actors constrained scalability, whereas facilitators included cultural adaptation, local leadership, and logistical support. Promoting ownership in highly mobile populations requires flexible designs, such as portable programs and training displaced lay health workers. Future research should focus on longitudinal studies and diverse crisis contexts to generate actionable insights, and contribute to more robust and adaptive healthcare approaches in crisis settings.

Overall, CHP interventions hold strong potential to bridge communities and health systems, but achieving equitable, sustainable, and adaptable outcomes will require investment in empowerment-based models, multisectoral collaboration, and innovative delivery strategies.

## Strengths and limitations

This scoping review offers several strengths, making it a valuable contribution to the understanding of participatory action research in crisis-affected settings. As the first of its kind, it comprehensively examines different types of NCD-focused CHP interventions applied in such contexts, providing a foundational understanding of multi-level community engagement across diverse populations. By incorporating a broad and inclusive approach, the review captures a wide range of interventions, ensuring diverse representation and highlighting key trends, gaps, and best practices. Stratifying studies according to levels of community participation provided a novel analytical lens, enhancing understanding of how engagement intensity influences outcomes. Methodological rigor was strengthened through a comprehensive multi-database search strategy, independent screening and data extraction by multiple reviewers with expertise in global health and humanitarian research, and transparent reporting of procedures. The breadth of included studies covered multiple conflict and displacement settings, increasing the contextual relevance of findings for humanitarian practice.

Despite its contributions, this review also has some limitations. The inclusion of only English-language studies may have led to the exclusion of relevant research published in other languages, thereby limiting geographic representation and affecting the generalizability of findings. However, given that over 90% of articles in major academic databases are published in English, the impact of this bias is likely limited. Additionally, the reliance on indexed databases means that unpublished research, grey literature, and community-led reports might not be fully captured, despite efforts to broaden the evidence base through Google Scholar searches. Furthermore, reliance on author-reported findings and, in some cases, single-statement outcome measures may have underestimated the strength or nuance of intervention effects. Another limitation stems from the strict inclusion criteria, which resulted in the exclusion of some high-quality participatory research studies that were not explicitly conducted in crisis settings, even though they could have provided valuable insights. Furthermore, the variability in study designs across the included researches makes it challenging to draw standardized comparisons or assess the overall effectiveness of interventions.

Despite these limitations, this scoping review provides a critical overview of participatory research in crisis-affected LMICs, identifying key challenges and opportunities for future research. By synthesizing a broad range of studies, it lays the groundwork for stronger community-driven interventions that can better address health challenges in crisis contexts; however, research is warranted to confirm and expand upon them.

### Implications for practice

Community-driven health interventions can bridge critical service gaps in humanitarian settings. Policymakers and implementing agencies should prioritize cultural adaptation, leverage trusted community actors, and invest in capacity-building for lay facilitators. Interventions that empower communities, rather than treating them as passive recipients, are more likely to gain traction, improve retention, and contribute to sustainable health outcomes.

### Implications for policy

Global and national health policies should institutionalize community participation frameworks as part of emergency preparedness and response strategies. Integration of CHP models into primary care systems and humanitarian action plans will be essential to achieve equity in health service delivery, especially for chronic NCD care. Donors should support long-term funding for participatory programs that emphasize ownership.

### Implications for future research

Future research should prioritize longitudinal designs to assess sustainability and long-term outcomes of CHP interventions. Standardized reporting of implementation processes – including barriers, facilitators, and participation levels – will strengthen the evidence base. There is also a need to expand research into underrepresented areas such as urban conflict zones, non-mental health NCDs, and high-mobility populations.

## Supplementary Material

Reporting guidelines_PRISMA_ScR Checklist_.docx

Supplementary _REVISED.docx

## Data Availability

Data can be shared upon request.
