# Supplementary material for: Community Health Participatory interventions in the prevention and control of non-communicable diseases including mental health in crisis-affected Low-and Middle-Income Countries – a scoping review
Source: Glob Health Action. 2026 Jan 2;19(1):2599011. doi: 10.1080/16549716.2025.2599011 (PMC12777845; doi:10.1080/16549716.2025.2599011)
Supplement: Supplementary _REVISED.docx [file ZGHA_A_2599011_SM8257.docx]

**SCOPING REVIEW**

**TITLE: Community Health Participatory interventions in the prevention and control of Non-communicable diseases in crisis affected Low-and-Middle-Income Countries- A scoping review**

**Supplementary files:**

**Supplementary Table 1: Operational definitions**

| **Terms** | **Operational definition** |
| --- | --- |
| Community | A community setting refers to a location or environment where people gather for purposes other than medical care. These settings are diverse and can include geographic communities, homes, schools, churches, homeless shelters, worksites, libraries, and more. The primary focus in such settings is typically on social, educational, religious, or other non-medical activities. [6] |
| Community Health Participatory (CHP) approach | CHP, a Community-Based Participatory Research (CBPR) approach in context of health, is a comprehensive research approach that encourages cooperation among community members, researchers, and various stakeholders to enhance health outcomes [12] |
| Non- communicable diseases | Non-communicable diseases (NCDs) refer to persistent health conditions that are not contagious, exhibit long-term duration, typically progress slowly, and are seldom completely curable. Some common NCDs that will be included in our scoping review include cardiovascular diseases (CVDs), cancers, chronic respiratory diseases, diabetes and mental illnesses. [1] |
| Low-and-Middle-Income Countries (LMICs) | As per the World Bank country classification for the fiscal year 2024, low-and-middle-income countries are characterised by a Gross National Income (GNI) per capita between $1,135 or less up to $13,845 as of 2022, including low income, lower-middle-income and upper-middle-income countries [3] |
| Crisis affected Area | Crisis affected areas refer to those areas, which are affected by war, political instability, or natural disasters that disturb vital services like housing, transportation, communication, sanitation, water, and healthcare. [8] |

**Supplementary table 2: Search Strategy**

The search strategy was developed as per the core themes of our research objectives, with iterative refinement guided by the University of York's librarian and operationalisation of definitions. Synonymous keywords were systematically explored in each database, utilising Boolean operators, truncation, proximity, and wildcard features tailored to the database's indexing criteria. The search strategy for PubMed is given below:

| PubMed:  ((((((((((((((((((("Community health participatory research"[Title/Abstract:~3]) OR ("Community-based participatory research"[Title/Abstract:~3])) OR ("action research"[Title/Abstract:~2])) OR ("participatory evaluation"[Title/Abstract:~2])) OR ("collaborative inquiry"[Title/Abstract:~2])) OR ("empowerment evaluation"[Title/Abstract:~2])) OR (mHealth[Title/Abstract])) OR ("participatory learning and action"[Title/Abstract:~3])) OR ("peer support"[Title/Abstract:~2])) OR ("community health"[Title/Abstract:~3])) OR ("participatory appraisal"[Title/Abstract:~2])) OR ("community based learning"[Title/Abstract:~2])) OR ("community engagement"[Title/Abstract:~2])) OR ("community group"[Title/Abstract:~2])) AND (("Noncommunicable diseases"[Title/Abstract:~2]) OR ("Non-communicable Chronic Diseases"[Title/Abstract:~3])) OR (Cardiovascular*[Title/Abstract])) OR (CVD[Title/Abstract])) OR (cancer*[Title/Abstract])) OR (asthma*[Title/Abstract])) OR ("chronic obstructive pulmonary disease"[Title/Abstract:~2])) OR ("chronic respiratory disorder"[Title/Abstract:~2])) OR (COPD[Title/Abstract])) OR ("diabetes mellitus"[Title/Abstract:~2])) OR ("type 2 diabetes"[Title/Abstract:~2])) OR (mental*[Title/Abstract])) OR (depression[Title/Abstract])) OR ("mental illness"[Title/Abstract:~2])) OR ("mental hygiene"[Title/Abstract:~2])) OR ("physical activity"[Title/Abstract:~2])) OR ("physical inactivity"[Title/Abstract:~2])) OR (exercise[Title/Abstract])) OR (walk*[Title/Abstract])) OR ("air quality"[Title/Abstract:~2])) OR (obesity*[Title/Abstract])) OR (overweight[Title/Abstract])) OR (obese[Title/Abstract])) OR (nutrition*[Title/Abstract])) OR (diet*[Title/Abstract])) OR (smok*[Title/Abstract])) OR (tobacco*[Title/Abstract])) OR (alcohol[Title/Abstract])) OR ("blood pressure"[Title/Abstract:~2])) OR (hypertension*[Title/Abstract])) OR ("chronic kidney disease"[Title/Abstract:~2])) OR (CKD[Title/Abstract])) OR (risk factor*[Title/Abstract]))) AND ((conflict*[Title/Abstract]) OR ("political instability"[Title/Abstract:~2])) OR (war[Title/Abstract])) OR ("informal settlement"[Title/Abstract:~2])) OR ("natural disaster"[Title/Abstract])) OR (man-made disaster*[Title/Abstract]:~2)) OR ("conflict affected"[Title/Abstract:~3])) OR ("internally displaced"[Title/Abstract:~3])) OR ("armed conflict"[Title/Abstract:~2])) OR ("post conflict"[Title/Abstract:~2])) OR (earthquake[Title/Abstract])) OR (flood[Title/Abstract])) OR (tsunami[Title/Abstract])) OR (drought[Title/Abstract])) OR (landslide[Title/Abstract])) OR (mudslide[Title/Abstract]))) AND ((afghanistan[Title/Abstract]) OR (afghanistan’s[Title/Abstract]) OR (afghanistani[Title/Abstract]) OR (afghani[Title/Abstract]) OR (afghan*[Title/Abstract]) OR (burundi[Title/Abstract]) OR (urundi[Title/Abstract]) OR (chad[Title/Abstract]) OR (congo[Title/Abstract]) OR (zaire[Title/Abstract]) OR (eritrea[Title/Abstract]) OR (ethiopia[Title/Abstract]) OR (gambia[Title/Abstract]) OR (korea*[Title/Abstract]) OR (liberia[Title/Abstract]) OR (malawi[Title/Abstract]) OR (madagascar[Title/Abstract]) OR (nyasaland[Title/Abstract]) OR (malaysia[Title/Abstract]) OR (maldives[Title/Abstract]) OR (mali[Title/Abstract]) OR (mozambique*[Title/Abstract]) OR (nigeria*[Title/Abstract]) OR (rwanda[Title/Abstract]) OR (sierra-leone[Title/Abstract]) OR (somali[Title/Abstract]) OR (sudan[Title/Abstract]) OR (syria[Title/Abstract]) OR (togo[Title/Abstract]) OR (uganda*[Title/Abstract]) OR (yemen*[Title/Abstract]) OR (angola[Title/Abstract]) OR (algeria*[Title/Abstract]) OR (bangladesh*[Title/Abstract]) OR (bengal*[Title/Abstract]) OR (bhutan[Title/Abstract]) OR (bolivia[Title/Abstract]) OR (cabo-verde[Title/Abstract]) OR (cape-verde[Title/Abstract]) OR (cambodia[Title/Abstract]) OR (cameroon[Title/Abstract]) OR (cameroun[Title/Abstract]) OR (comoro[Title/Abstract]) OR (comore[Title/Abstract]) OR (djibouti[Title/Abstract]) OR (egypt*[Title/Abstract]) OR (eswatini[Title/Abstract]) OR (swaziland[Title/Abstract]) OR (swati*[Title/Abstract]) OR (ghana[Title/Abstract]) OR (guinea[Title/Abstract]) OR (haiti[Title/Abstract]) OR (hondura[Title/Abstract]) OR (jordan*[Title/Abstract]) OR (india*[Title/Abstract]) OR (iran*[Title/Abstract]) OR (kenya*[Title/Abstract]) OR (kiribati[Title/Abstract]) OR (kyrgyz*[Title/Abstract]) OR (lebanon[Title/Abstract]) OR (lebanese[Title/Abstract]) OR (lesotho[Title/Abstract]) OR (mauritania[Title/Abstract]) OR (mauritan[Title/Abstract]) OR (mauritius[Title/Abstract]) OR (micronesia[Title/Abstract]) OR (mongol[Title/Abstract]) OR (morocco[Title/Abstract]) OR (moroccan[Title/Abstract]) OR (myanmar[Title/Abstract]) OR (burma[Title/Abstract]) OR (nepal*[Title/Abstract]) OR (nicaragua[Title/Abstract]) OR (pakistan*[Title/Abstract]) OR (philippine[Title/Abstract]) OR (philipine[Title/Abstract]) OR (phillipine[Title/Abstract]) OR (samoa[Title/Abstract]) OR (senegal[Title/Abstract]) OR (solomon-island[Title/Abstract]) OR (sri-lanka*[Title/Abstract]) OR (tajik*[Title/Abstract]) OR (tanzania[Title/Abstract]) OR (timor-leste[Title/Abstract]) OR (tunisia[Title/Abstract]) OR (ukrain*[Title/Abstract]) OR (vanuatu[Title/Abstract]) OR (vietnam[Title/Abstract]) OR (viet-nam[Title/Abstract]) OR (zambia[Title/Abstract]) OR (zimbabwe[Title/Abstract]))) |
| --- |

**Supplementary Table 3: Study details and target population**

| **Author (Year of Publication)** | **Name of Intervention** | **Area of Research** | **Type of crisis** | **Country** | **Target Population** | **Purpose of intervention** |
| --- | --- | --- | --- | --- | --- | --- |
| Akhtar et al. (2021)[26] | Scalable Transdiagnostic Psychological Intervention (PM+) | Mental Health | Civil War | Jordan | Refugees with mental health disorder (and had a child aged 10-16) | Reduction of psychological distress in refugee parents and children. |
| Bogdanov (2021)[27] | Community-Based Transdiagnostic Psychotherapy | Mental Health | Ongoing military conflict | Ukraine | Veterans and internally displaced persons | Reductions in depression, anxiety, posttraumatic stress symptoms among conflict affected adults and army veterans in Ukraine |
| JordansMJD et al. (2021)[28] | Group Problem Management Plus | Mental Health | Humanitarian disasters (floods, civil war) | Nepal | Adults with psychological distress | Reduction in psychological distress and functional impairment through trained facilitators who did not receive any prior professional mental health training |
| Musisi et al. (2021)[29] | Psychological First Aid (WHO) | Mental Health | Humanitarian crisis (Sucide, domestic violence, food shortage) | Uganda | Population residing in post- conflict rural communities | To provide community based psychological/mental support through trained volunteer village health teams |
| Lee et al. (2022)[30] | Psychosocial Support Focal Point Response | Mental Health | Covid-19 Pandemic | Myanmar | Internally displaced population camps | To provide psychosocial support to a vulnerable Internally displaced population in the midst of the COVID-19 pandemic |
| Agiresaasi et al. (2022) [31] | Alcohol Use Communication Intervention | Alcohol intake | Civil War | Uganda | Women in their reproductive age | Reduction in alcohol use during pregnancy through a communication intervention |
| Mphekgwana et al. (2022)[32] | Community Action Model for NCDs | Cardiovascular Disease (CVD) | Humanitarian disasters (floods, civil war) | South Africa | Community members at moderate risk of developing CVD | Reduce risk for CVD and metabolic syndromes |
| Augustinavicius et al. (2023)[33] | Self-Help Plus (SH+) | Mental Health | Ongoing military conflict | Uganda | Refugee Women | Reduction in mental disorders, with increased positive mental health and well-being |
| Rattner et al. (2023)[34] | Community-Based Psychosocial Support (CB-PSS) | Mental Health | internal armed conflict exacerbated by Covid-19 Pandemic | Columbia | Adults affected by violence | Reduction in symptoms of generalized distress, anxiety, depression, and posttraumatic stress. |
| Paphitis et al. (2023)[35] | Counseling on Wheels for Mental Health & Peacebuilding | Mental Health | Humanitarian crisis (Boko Haram conflict) | Nigeria | All adult community members | Reduction in depression, stress, anxiety and vulnerability to violent extremism |
| Khan et al. (2017)[36] | WHO trans-diagnostic intervention - Group PM+ | Mental Health | Military armed conflict | Pakistan | Women with mental disorder living in conflicted areas | Reduction in psychological distress, depression and levels of functioning through trained lay helpers |
| Sethi et al. (2017)[37] | Community-Based NCD Care | NCDs | Civil War | Lebanon | Refugees | Delivery of NCD care through community-based primary health activities via volunteer refugee health workers |
| Becker et al. (2009)[38] | Psychosocial Care Initiative | Mental Health | Tsunami Disaster (Floods) | India | Women affected by the disaster | Delivery of psychological care to women tsunami survivors |
| Sangraula (2020)[39] | WHO Group Problem Management Plus (PM+) | Mental Health | Earthquake | Nepal | Adults with mental health disorder | Delivery of psycho-education to reduce mental health issues |
| Welton-Mitchell et al. (2018)[40] | Mental Health & Disaster Preparedness | Mental Health | Earthquake | Nepal | Adult Community members affected by the disaster | Improving disaster preparedness, mental health, and community cohesion |
| James et al. (2020)[41] | Mental health and disaster preparedness intervention | Mental Health | Earthquake and flood | Haiti | Communities affected by the disaster | Improving disaster preparedness and reduction in symptoms associated with depression, post-traumatic stress disorder, anxiety, and functional impairment, and increased peer-based help-giving and help-seeking |
| Vijayakumar et al. (2017)[42] | Suicide Prevention (CASP) | Mental Health | Military armed conflict | India | Refugee Camps (suicidal or depressed individuals) | To reduce suicidal behaviour among Sri Lankan refugees |
| Khan et al. (2017)[43] | Local Psychoeducation Intervention | Mental Health | Military armed conflict | Pakistan | Pregnant Women with psychological distress | Reduction in mental health issues such as psychological distress |
| Rahman (2019)[44] | A brief psychological intervention- Group PM+ | Mental Health | Military armed conflict | Pakistan | Women with mental health disorder | To reduce symptoms of anxiety and depression |
| Tamasese et al. (2020)[45] | Asiasiga: a Samoan intervention to address the immediate mental health needs | Mental Health | Tsunami Disaster (Floods) | Samoa | Families affected by the disaster | To address the mental health needs of affected communities via Samoan culture practice |
| Scholte et al. (2011)[46] | Psychosocial Sociotherapy | Mental Health | Civil War | Rwanda | Adults affected by mass violence | Improvement in mental health and social bonding |
| Khoja (2016)[47] | Conventional & Telehealth Mental Health Solutions | Mental Health | Military armed conflict | Afghanistan | Community Members and young adults | Reduction in symptoms of depression, psychosis, post-traumatic stress disorder, and decreased substance abuse. |
| Krishnaswamy et al. (2012)[48] | Early Mental Health Intervention | Mental Health | Tsunami floods | Malaysia | Adults affected by the disaster | To address mental health issues and provide help to prevent the mental health problems |
| Erenoğlu et al. (2020)[49] | Breast & Cervical Cancer Education | Cancer | Civil War | Turkey | Refugee Women | Increase awareness of breast and cervical cancer in women |
| Verduin et al. (2014)[50] | Sociotherapy for Social Capital | Mental Health | Civil War | Rwanda | Adults residing in post conflict areas | Improve mental health and social capital through sociotherapy |
| Sanhori (2019)[51] | Mental Health Stigma Awareness | Mental Health | Drought, famine and civil war | Central Sudan | Internally displaced population camps | Reduce mental health stigma among participants |

**Supplementary Table 4: Intervention outcomes**

| **Author (Year of Publication)** | **Name of Intervention** | **Tools used** | **Key Outcomes of intervention** |
| --- | --- | --- | --- |
| Akhtar et al. (2021)[26] | Scalable Transdiagnostic Psychological Intervention (PM+) | WHODAS 2.0 , K10. | Culturally acceptable and safe intervention with High retention rates |
| Bogdanov (2021)[27] | Community-Based Transdiagnostic Psychotherapy | Self-administered digital survey on CommCare | Reduction in symptoms of depression, anxiety and functional impairment. |
| JordansMJD et al. (2021)[28] | Group Problem Management Plus | Psychological distress assessed with the General Health Questionnaire (GHQ-12), Patient Health Questionnaire (PHQ-9), PTSD CheckList (PCL), Multidimensional Scale of Perceived Social Support (MSPSS), Somatic Symptom Scale 8 (SSS-8), and WHODAS-II | Reduction in psychological distress and symptoms of depression |
| Musisi et al. (2021)[29] | Psychological First Aid (WHO) | SRQ-20 used to study psychological distress, WHO-mhGAP, Psychological First AID (PFA) manual | Improved dealing with conflicts with the help of social support |
| Lee et al. (2022)[30] | Psychosocial Support Focal Point Response | Evaluation questionnaire, analysis of the focal point logs | High reach (93%) and acceptability, usefulness of intervention handouts, recognition of mental health counselling services to seek help and attain information |
| Agiresaasi et al. (2022) [31] | Alcohol Use Communication Intervention | WHO WHS (World Health Survey), WHO Alcohol Use Disorder Identification Test (AUDIT) | Reduction in alcohol consumption and dependence. |
| Mphekgwana et al. (2022)[32] | Community Action Model for NCDs | The non-laboratory INTERHEART Risk Score tool. | Increased CHWs knowledge on CVD screening and prevention. Improved behavioural practices for NCD risk factors |
| Augustinavicius et al. (2023)[33] | Self-Help Plus (SH+) | K6, WHODAS, PCL-6,  PHQ-9, WHO-5, AAQ-II | Lower probable mental disorder in intervention group compared to control |
| Rattner et al. (2023)[34] | Community-Based Psychosocial Support (CB-PSS) | Qualitative assessments | Participants reported overall high satisfaction with increased social support and group cohesion. Accessibility and security of the spaces used during in-person sessions were highly valued in both in-person and hybrid modalities. |
| Paphitis et al. (2023)[35] | Counseling on Wheels for Mental Health & Peacebuilding | Depression, Anxiety and Stress Scale (DASS-21),  Post-Traumatic Stress Disorder Scale (PTSD-8),  Vulnerability to Violent Extremism Scale (VVES) | Scores for depression, stress and vulnerability to violent extremism were reduced |
| Khan et al. (2017)[36] | WHO trans-diagnostic intervention - Group PM+ | Hospital anxiety & depression rating scale(HADS), Hospital anxiety rating scale, Hospital depression rating scale,  WHO DAS,  Psychological outcome profiles (PSYCHLOPS), PCL, PHQ-9, Process evaluation | Reduction in HADS score, 61% participants showed consistence attendance in all 5 sessions, considered helpful and satisfactory by participants and families. |
| Sethi et al. (2017)[37] | Community-Based NCD Care | Knowledge, Practice and Coverage (KPC) was used to assess the prevalence of NCDs, Risk Factors and access to Health Service | Multiple successful monitoring visits, referrals, and empowerment of community through training of 500 community outreach volunteers |
| Becker et al. (2009)[38] | Psychosocial Care Initiative | Self-Reporting Questionnaire (SRQ) -to assess the level of mental disability, IES16 (Impact of Event Scale) - to measure emotional distress | Reduction in IES scores- avoidance, intrusion, hypervigilance |
| Sangraula (2020)[39] | WHO Group Problem Management Plus (PM+) | Patient Health Questionnaire (PHQ-9),  WHO Disability Assessment Scale (WHODAS), GHQ-12, The Psychosocial Mental Health Problems (PMHP),  PCL-5, Multidimensional Scale of Perceived Social Support (MSPSS),  The Reduced Tension Checklist (RTC),  Psychological Outcomes Profiles instrument, | Study showed feasibility and acceptability to deliver intervention through non-specialists. |
| Welton-Mitchell et al. (2018)[40] | Mental Health & Disaster Preparedness | Adapted versions of Humanitarian Emergency Settings Perceived Needs (HESPER),Patient Health Questionnaire (PHQ-9),Posttraumatic Stress disorder (PTSD) Checklist – Civilian Version (PCL-C), and qualitative evaluations | Study reported increased resilience in earth-quack affected communities |
| James et al. (2020)[41] | Mental health and disaster preparedness intervention | Qualitative assessment | Increased disaster preparedness and social cohesion, and reduction in mental health symptoms |
| Vijayakumar et al. (2017)[42] | Suicide Prevention (CASP) | Survey, focus group discussion | Demonstrated feasibility of implementation and reduction of suicidal behaviour among refugee participants. |
| Khan et al. (2017)[43] | Local Psychoeducation Intervention | WHO-mhGAP, SRQ used to assess Psychological distress, Multidimensional Scale of Perceived Social Support (MSPSS) to assess social support | Feasible delivery of intervention with good acceptance, with 71% women (intervention arm ) contacting LHWs for assistance within 2 months of intervention. |
| Rahman (2019)[44] | A brief psychological intervention- Group PM+ | Hospital Anxiety and Depression Scales (HADS) | Significant reduction in HADS score, High competency among facilitators, 82% participants attending 3 or more sessions. |
| Tamasese et al. (2020)[45] | Asiasiga: a Samoan intervention to address the immediate mental health needs | Feedback from respondents | High degree of acceptance from families, and upon request a children’s programme was also launched |
| Scholte et al. (2011)[46] | Psychosocial Sociotherapy | Mental health was assessed using the Self Reporting Questionnaire (SRQ-20) | Reduction in SRQ-20 scores showing improved mental health |
| Khoja (2016)[47] | Conventional & Telehealth Mental Health Solutions | Surveys, follow-up interviews, focus group discussions,  evaluation of the use of mental health guidelines and referrals by healthcare providers | Improved access to care, reduced stigma towards mental health and better quality of health services. |
| Krishnaswamy et al. (2012)[48] | Early Mental Health Intervention | Clinical Interview Schedule–Revised (CIS-R) instrument | Stabilized mental health among victims of tsunami. |
| Erenoğlu et al. (2020)[49] | Breast & Cervical Cancer Education | Self-structured questionnaire | Increased awareness of breast and cervical cancer |
| Verduin et al. (2014)[50] | Sociotherapy for Social Capital | SRQ-20 for screening common mental disorders, Adapted Social Capital Assessment Tool, (Short A-SCAT) for measuring social capital | Increased civic partnership and mental wellbeing. |
| Sanhori (2019)[51] | Mental Health Stigma Awareness | Standardized stigma attitude tools prepared by Word Psychiatric Association | No change in attitudes and stigma towards mental health. |
